# Methods of identifying and recruiting older people at risk of social isolation and loneliness: a mixed methods review

**DOI:** 10.1186/s12874-019-0825-6

**Published:** 2019-08-29

**Authors:** Janet Ige, Lynn Gibbons, Issy Bray, Selena Gray

**Affiliations:** 1 Centre for Public Health and Wellbeing, University of the West of England, Bristol, Frenchay Campus, Bristol, BS16 1QY UK; 2 South Gloucestershire Council, Yate, UK

**Keywords:** Loneliness, Social isolation, Recruitment, Older adults

## Abstract

**Background:**

Loneliness and social isolation are major determinants of mental wellbeing, especially among older adults. The effectiveness of interventions to address loneliness and social isolation among older adults has been questioned due to the lack of transparency in identifying and recruiting populations at risk. This paper aims to systematically review methods used to identify and recruit older people at risk of loneliness and social isolation into research studies that seek to address loneliness and social isolation.

**Methods:**

In total, 751 studies were identified from a structured search of eleven electronic databases combined with hand searching of reference bibliography from identified studies for grey literature. Studies conducted between January 1995 and December 2017 were eligible provided they recruited community living individuals aged 50 and above at risk of social isolation or loneliness into an intervention study.

**Result:**

A total of 22 studies were deemed eligible for inclusion. Findings from these studies showed that the most common strategy for inviting people to participate in intervention studies were public-facing methods including mass media and local newspaper advertisements. The majority of participants identified this way were self-referred, and in many cases self-identified as lonely. In most cases, there was no standardised tool for defining loneliness or social isolation. However, studies that recruited via referral by recognised agencies reported higher rates of eligibility and enrolment. Referrals from primary care were only used in a few studies. Studies that included agency referral either alone or in combination with multiple forms of recruitment showed more promising recruitment rates than those that relied on only public facing methods. Further research is needed to establish the cost-effectiveness of multiple forms of referral.

**Conclusion:**

Findings from this study demonstrate the need for transparency in writing up the methods used to approach, assess and enrol older adults at risk of becoming socially isolated. None of the intervention studies included in this review justified their recruitment strategies. The ability of researchers to share best practice relies greatly on the transparency of research.

## Background

Loneliness and social isolation have been identified as having significant impacts on the health and wellbeing of older people. The effect of this is not limited to increased risk of developing chronic diseases, but also includes mental health conditions and high dependency on primary and secondary care services, as summarised in a recent review by Goodman and colleagues [1].

There is increasing recognition of the prevalence and importance of social isolation as a public health issue in the UK. This has led to the development of a wide range of programmes and interventions by the public sector and voluntary organisations. Examples include A Campaign to End Loneliness [2] and The Big Lottery Better Ageing Programme [3]. Social isolation indicators - albeit for those already in contact with social services - are now included in the Public Health Outcomes Framework. While there is an emerging evidence base on the strengths and weaknesses of different measurement tools to define loneliness and social isolation [2], it is still not clear what methods are best used in practice to identify those who are (or may be at risk of being) socially isolated or lonely. Hidden Citizens, A Campaign to End Loneliness consultation, highlighted the need for an identification and recruitment tool to be used by frontline providers who provide support for older people [2]. This difficulty is also reflected in the research community, where little information has been published regarding the methods used for the specific identification and recruitment of older people who may be considered at risk of social isolation and loneliness.

Although there has been research into the recruitment of older people into clinical trials linked to specific morbidities [4], there is little published research regarding methods of community-based recruitment into research studies on loneliness and social isolation. A systematic review by Age UK conducted to provide information for commissioners and clinicians about schemes to alleviate loneliness, highlighted that participation in such programmesmes is often low, that ‘identifying people at risk of loneliness can be difficult’ (p.3) and that older men, in particular, are ‘notoriously hard to reach’ (p. 26). It also highlighted the importance of assessing the effectiveness of individual schemes [5].

In the last 10 years, three systematic reviews of interventions to reduce and prevent loneliness have been undertaken [6–8]. These were reviews of interventions only and did not have a focus on investigating the methods used in the identification and recruitment of participants into such studies. The authors of this paper are not aware of any review to date with an emphais on identification and recruitment. The aim of this paper is, therefore, to systematically review the methods used to identify and recruit older people into research studies aimed at tackling loneliness and social isolation in the community. Available data on factors associated with success and barriers to recruitment will also be assessed. Finally, it will examine whether specific methods are more successful with particular subgroups of the population (e.g., by gender or ethnicity).

## Methods

### Searches

Eleven electronic databases were searched for eligible studies - AMED, CINAHL, MEDLINE, PsychINFO, SOCINDEX, EMBASE, Social Practice & Policy, JSTORE (health, public health, and social sciences), ASSIA (Applied Social Sciences Indexes and Abstracts), SOCABS and Social Care Online. The literature search was undertaken by two researchers (LG and JI) with support from a subject librarian. Search terms were grouped by topic and included those linked to the participants (older adults), the exposure (loneliness and isolation) and outcomes (Table 1). Reference lists were used to identify any additional articles that had not been picked up by the search strategy. Also, grey literature, including publications by relevant voluntary sector organisations, was searched for any additional articles referenced.

**Table 1:** Search terms

Participants	Exposure	Outcome
Old*	Isolat*	Combat*
Senior*	Lone*	Tackl*
Elder*	Alone*	Reduc*
OAP	Social N2 exclus*	Decreas*
Aged	Social N2 alienat*	Lessen*
Aging	Bereave*	Preven*
Mature		Support*
Geriatric		Intervent*
Retire*		

The search terms for each column was combined with OR. The result from each column was then combined with AND

### Inclusion and exclusion criteria

Studies were included if they met the following criteria: discuss, evaluate or pilot an intervention which aimed at reducing loneliness or social isolation; include community living individuals aged 50+. Studies were required to be published in English between 1995 and 2017 and conducted in any high-income country according to the World Bank classification [9].

Studies that included people less than 50 years old were excluded. Also excluded were studies set in countries other than those stated above due to differences in the cultural and social support for and responses to older people in the community. Studies which were part of, or used data collected from, an existing larger study (e.g., a population cohort study), were not included because these were not recruiting directly from the community, and thus the methods used would not be replicable in practice or an intervention study.

### Data extraction

Data extraction was conducted by two reviewers (JI and LG) using a standardised data extraction sheet created to record details of the author, study design, location, target population and methods of recruitment, the number of participants approached, the number of participants assessed for eligibility, the number of eligible and willing participants recruited. Where available, information on dropout rate and cost of recruitment were extracted. The standardised data extraction tool was tested by JI and LG in discussion with the other authors. Since the purpose of the review was not to summarise the effectiveness of the interventions, the studies were not formally assessed for quality.

## Results

**Fig. 1:**
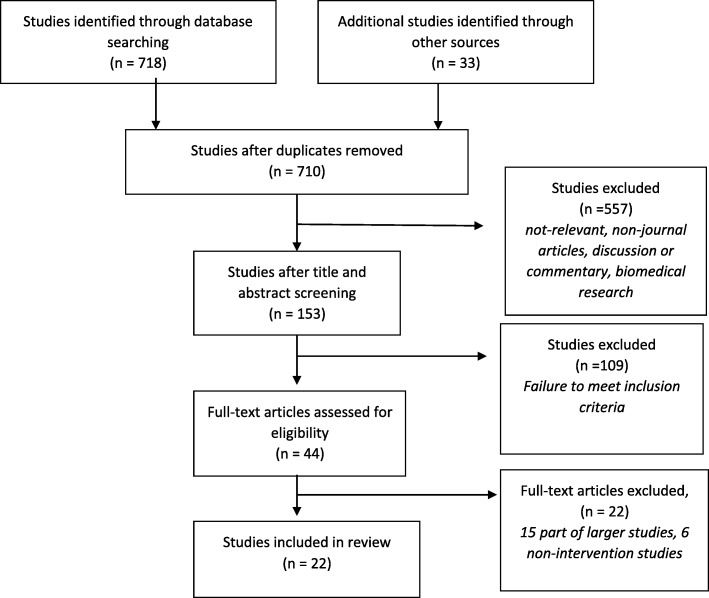
Study selection process

The database search returned a total of 718 studies. Additional studies (*n* = 33) were identified through Google Scholar citations, hand searching of reference list of relevant studies and cross-referencing. Following the removal of 41 duplicates, 708 studies were screened by title and abstract; this led to further removal of 557 studies. The full text from 44 studies was assessed against the inclusion and exclusion criteria, and based on this, 22 studies were further excluded. The majority of the excluded studies obtained data from national cohort studies and did not have a focus on isolated or socially excluded older adults. A final selection of 22 studies were included in this review. The result of the search is summarised in Fig. 1 below.

The included studies were conducted between 2000 and 2017. Although the studies differed in many aspects, including the type of intervention, they all shared a broadly similar goal, which was to either pilot an intervention or evaluate the effectiveness of an intervention to prevent social isolation and loneliness among older adults. In terms of the study design, ten out of the 21 studies were RCTs; six were quasi-experimental studies, two were qualitative studies, while the remaining four studies were identified as a prospective controlled trial, mixed method study, before and after study and a longitudinal study. The majority of the studies (*n* = 8) were conducted in the US, six studies were conducted in The Netherlands, three in Finland and two in the UK. The remaining three studies were conducted in Canada, Australia, and Japan respectively. The number of participants varied considerably and ranged from 17 to 858 participants.

The interventions were delivered across various settings but mainly in community settings (*n* = 14). Some interventions were delivered at homes (*n* = 2), homes and community (*n* = 4), or community, homes, and nursing home (*n* = 4). Five of the studies introduced the internet [10–13] or Nintendo Wii [14] as a key component of the intervention. Nine studies featured multiple interventions, where at least one of the interventions involved supportive work in groups [15–23]. Three other studies included interventions focussed on individual programmes such as mentoring [24], mindfulness [25] and befriending [26]. One study delivered a 6 weeks course on friendship via the internet [27]. Further characteristics of included studies are presented in Table 2 below.

**Table 2:** Characteristics of included studies

Author, year	Location	Type of study	Aim of study	Intervention setting
Bouwman et al., 2017	The Netherlands	Quasi-experimental	To evaluate the effectiveness of an online adaptation of the friendship enrichment programme	Community
Collins and Benedict, 2006	USA	Pre-experimental, pre-test, post-test	To evaluate the effects of a community-based educational program designed to promote health by enhancing older adults’ mastery while decreasing loneliness and stress	Community
Cox et al., 2007	USA	Quasi-experimental	To increase the capacity of cognitively able elders to effectively manage their own care and optimize relationships with caregivers	Community, home and nursing home
Cresswell et al., 2012	USA	RCT	To test the effectiveness of an 8-week mindfulnes stress reduction program on loneliness among older adults	Community
Dickens et al., 2011	UK	Prospective Controlled Trial	To evaluate the effectiveness of a community-based mentoring service for improving mental health, social engagement and physical health for socially isolated people	Community
Freidman et al., 2017	USA	Pre and post intervention	To pilot test an intervention aimed at promoting psychological well-being among community living older adults	Community
Fokkema and Knipscheer, 2007	Netherlands	Mixed method- qualitative and quantitative study	To evaluate the outcome of a digital intervention to reduce loneliness among chronically ill and disabled older adults	Home
Gracia and Moyle, 2010	Australia	Qualitative	To assess perceptions of a self-help print-delivered intervention aimed at addressing loneliness in a retirement village community.	Retirement village
Greenwald and Beery, 2001	USA	Longitudinal study design	To evaluate the outcome of an intervention designed to reduce isolation and improve quality of life	Community
Honigh-De Vlaming et al., 2013a	Netherlands	Qualitative study	To investigate how mass media communication materials, information meetings and psychological courses were received by elderly people at high risk of isolation	Community
Honigh-De Vlaming et al., 2013b	Netherlands	Quasi-experimental pre-test post-test	Evaluation of a two-year complex intervention to reduce loneliness in non-institutionalised elderly Dutch people	Home and group
Jones et al., 2015	UK	Before and After	To evaluate the value and impact of internet use on loneliness among older people	Home and group setting
Kahlbaugh et al., 2011	USA	RCT	To investigate the effect of Wii technology on physical activity, loneliness and mood.	Home
Kremers et al., 2006	Netherlands	RCT	Effect of a short group intervention on self-management, well-being, social and emotional loneliness of single older women	Community
McAuley et al., 2000	USA	RCT	To evaluate the effect of physical activity on subjective wellbeing (SWB) and the role played by physical activity participation and social support in changes in SWB over time	Community
Ollonqvist et al., 2008	Finland	RCT	To determine the effectiveness of a new rehabilitation model on loneliness among frail older people	Rehabilitation centres
Pitkala et al., 2009	Finland	RCT	To investigate the impact of group rehabilitation on use of health care services and mortality from older persons suffering from loneliness (same population as Routasalo et al., 2009)	Rehabilitation centre
Routasalo et al., 2009	Finland	RCT	To evaluate the effect of a psychosocial group nursing intervention on older people’s feelings of loneliness, social activity and psychological well-being (same population as Routasalo)	Rehabilitation centre
Saito et al., 2012	Japan	RCT	To investigate the effect of a social isolation prevention program on loneliness, depression and well-being of older adults in Japan	Community
Slegers et al., 2008	Netherlands	RCT	To investigate the relationship between computer use and wellbeing (physical, social and emotional) and quality of life	Community and home
Stewart et al., 2001	Canada	Pre-test, post-test	To evaluate the impact of support groups on widowed seniors’ loneliness/isolation.	Community
White et al., 2002	USA	RCT	To evaluate the impact of providing internet training and access to older adults	Community and nursing home

### Method of recruitment

A range of strategies was used to recruit participants to the intervention (Table 3). The most common ones were print media, information sessions, referral, e.g. by General Practice (GP) and a combination of one or more of these strategies. One study did not provide any information on how participants were recruited [23].

**Table 3:** Method of recruitment

Author/ year	Methods of recruitment
Bouwman et al., 2017	Online advertisement, newspaper advertisements across specific regions
Collins and Benedict, 2006	Promotional flyers and newsletters at chosen senior centres and senior housing developments
Cox et al. 2007	Agencies, seniors’ groups and churches
Cresswell et al. 2012	Newspaper advertisements from local area
Dickens et al. 2011	Participants for the intervention group were recruited from cohort of individuals currently in receipt of mentoring while control group participants were recruited from those receiving usual care
Freidman et al., 2017	Participants were referred by community service organisations, others were recruited through newspaper advertisement and information flyers
Fokkema and Knipscheer 2007	Purposively selected by volunteer home visitors of Red Cross and disability support charity
Gracia and Moyle, 2010	Information was sent to managers of retirement village
Greenwald and Beery 2001	Community specialists identified individuals isolated or at risk of isolation through community agencies, food banks and city housing authority. Matched individuals throughout central and SE Seattle.
Honigh-De Vlaming et al. 2013a	Invitation letter sent to 250 clients together with their meals. Of the 250 invitation letter, 14 were returned with an interest to participate, 3 partners were also included in the study (*n* = 17)
Honigh-De Vlaming et al. 2013b	A range of approaches (mass-media campaign, stand at municipal information fair, monthly article in local newspaper, municipal information booklets, posters and brochures
Jones et al. 2015	Beneficiaries – awareness and referral from Age UK. Awareness raised through tenants of Plymouth Community Homes, adverts in community newspapers and bus shelters, attendance at local events and personal contacts. Volunteers were recruited via local advert.
Kahlbaugh et al. 2011	Participants were recruited through flyers posted in residential facilities and through informational sessions
Kremers et al. 2006	Advertisements in local newspapers in two regions of the Netherlands.
McAuley et al. 2000	Range of recruitment techniques – local newspaper, announcement and infomercials on local TV and radio, flyers in grocery stores, churches, senior centres
Ollonqvist et al. 2008	Selection by local social and health service staff from 7 independent rehabilitation centres & 41 municipalities
Pitkala et al. 2009	Postal questionnaire sent to a random sample of older people in 6 communities from the Finnish National Population register. The initial questionnaire required respondents to self-identify if they suffered from loneliness. a consent form was sent to those who self-identified
Routasalo et al. 2009	Same as Pitkala et al. (2009)
Saito et al., 2012	Respondents were identified from the basic resident registration card
Slegers et al. 2008	Flyer were randomly sent to 64–75 year olds on the city register
Stewart et al. 2001	No information on how the women were recruited
White et al. 2002	Information sessions for residents at housing and leaflets/flyers in housing

#### Print media

Five studies reported using a variety of printed materials including flyers, information sheets, and newspaper adverts to recruit participants to their intervention [11, 15, 17, 19, 25]. These materials were posted in many different public locations including senior centres and senior housing developments [15]. Honigh-De Vlaming and colleagues distributed invitation letters together with the meal of clients on a meal delivery service of local elderly welfare [17]. In the study by Slegers and colleagues, flyers were sent to 6054 older adults in a residential area [11] while Kremers et al. [19] and Cresswell et al. [25] advertised in local newspapers.

#### Referral

Six studies recruited participants via referral from a number of different agencies including community teams and GP practices [24], charity organisations [13], seniors’ groups and churches [16], social health service staff [20], housing authority and food banks [26] and managers of retirement villages [28].

#### Multiple methods of recruitment

Eight studies employed a combination of two or more strategies to recruit participants. In two of the studies, participants were recruited via newspaper advertisement, information flyers, and referral by organisations and agencies [10, 18]. Two other studies recruited participants via information sessions and flyers posted in residential facilities [12, 14]. One study combined online advertisement on a community website with newspaper advertisement while another study recruited participants via infomercials on local TV and print media in the form of newspaper advertisement, flyers in grocery stores, churches and senior centres [29]. One study recruited participants via mass media campaign, attendance of information fair, newspaper articles, booklets, posters, brochures and GP referral [30].

#### Other methods of recruitment

In two studies, letters and questionnaires were sent to a random sample of older adults identified from a national population register [21, 22]. One study identified participants from a basic registration card [31]. Table 3 below details the method of recruitment for each of the identified studies.

### Target population

Four studies did not report any age related eligibility criteria [12, 14, 17, 28]. Three other studies identified participants as ‘older’ or ‘elderly’ without providing any age benchmark [13, 15, 26]. Of those that specified the age related eligibility criteria, the definition of ‘older’ ranged between 50+ [25, 27], 55+ [16, 19, 23, 25], 60+ [18, 29],, 64+ [11], 65+ [10, 20, 30, 31] and 75+ [21].

The majority of the studies described their target audience as healthy or independent older adults. One study enrolled participants who received a minimum of 6 h of personal care/week and reported one or more pre-defined disability [16]. Participants in Dickens et al. were required to be identified by community teams as socially isolated or at risk of social isolation [24]. However, as with most of the included studies, there wasn’t a clear definition of social isolation. Another study enrolled non-personal computer users who lived alone and had few possibilities to leave their home [13]. One study enrolled widows who had not engaged with any support group [23].

### Identification of respondents

The methods for identifying eligible participants was vague in many studies; 13 studies did not discuss any standardised tool for self-referrals and relied on participant’s self-declaration of loneliness or social isolation [11, 14, 15, 17, 19, 21–23, 25, 26, 28–31]. Only eight studies reported enrolling participants highlighted by professionals to be at risk of, or suffering from, loneliness or social isolation [10, 12, 13, 16, 18, 20, 25, 26]. In all of these studies, referrals were made from front-line statutory and voluntary services such as housing support and social care, and only two used referrals from primary care within GP practices [20, 24]. Two studies enrolled participants who either self-referred or were selected by individuals or organisations including healthcare professionals and local agencies [10, 12].

The authors of the studies that enrolled participants via self-referral provided very little information regarding formal assessment of eligibility. Three studies enrolled participants who answered ‘yes’ to a loneliness question on a questionnaire ([21, 22]), one study recruited participants from the general population, and then used a loneliness scale at baseline and follow up to determine loneliness [27]. One included those who were interested in learning mindfulness [25]. Of those that used referrals from other services, there was also limited information on the assessment of eligibility and quantification of social isolation and loneliness. In general, inclusion was based on age, willingness to take part, cognitive ability, and living arrangements, i.e., living alone.

### Uptake and dropout rate

#### Total number of participants approached/referred

Half of the studies (*n* = 11) did not report the number of respondents referred or approached [10, 12–16, 19, 20, 23, 25, 26]. For the remaining studies, the number of participants referred/approached ranged from 134 to 6786.

#### Number of participants assessed for eligibility

The number of participants assessed for eligibility varied greatly and ranged from 22 to 3871. There was a considerable disparity between the number of participants approached/referred and the number of participants assessed for eligibility. In the study by Slegers and colleagues, 6054 participants were approached while only 1016 of them (17%) expressed interest in the study and were assessed for eligibility [11]. In the same manner, Saito et al. approached 709 participants out of which 76 (11%) participants were assessed for eligibility [30]. Honigh-De Vlaming et al. approached 2718 participants and received interest from 1804 (66%) of them [30]. Six studies did not provide information on the number of participants assessed for eligibility [14, 17, 18, 20, 28, 29].

#### Number of eligible and willing participants recruited

**Fig. 2:**
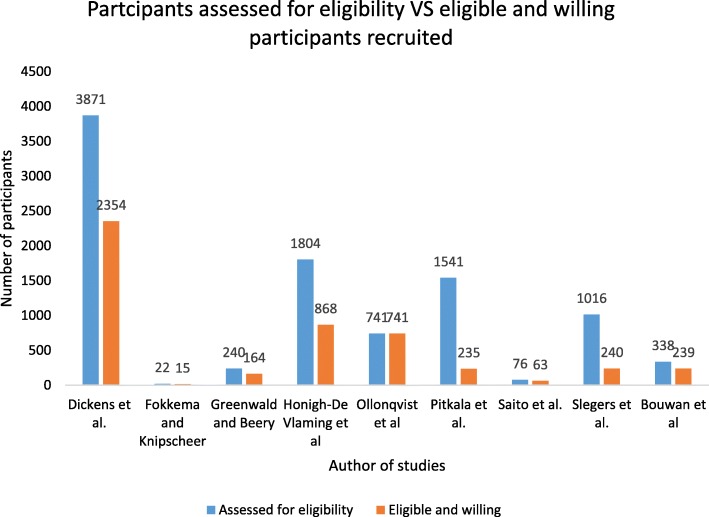
Number of participants assessed for eligibility and number of eligible and willing participants recruited (*n* = 8 studies provided sufficient data to calculate this)

The number of eligible participants who were willing to be recruited into the studies ranged from 15 [13] to 2535 [24]. There was a considerable variation between the number of participants assessed for eligibility and the number of eligible and willing participants recruited. The study by Bouwan et al. (2017) reported that 239 of 338 participants who had indicated an interest in the study, completed baseline assessments. The graph below (Fig. 2) shows the difference in the number of participants assessed for eligibility and the actual number of eligible and willing participants recruited. One study did not report on the number of eligible and willing participants recruited [16].

Overall, there was little information provided on the challenges involved in identifying and determining the eligibility of participants. There was also no indication as to why specific recruitment methods were chosen or if other methods were discarded. Those using referrals from statutory agencies did not detail any tools and training for staff, to enable them to identify individuals at risk appropriately. There was insufficient information about the demographics of participants and the wider population. Generally, the study sample for most of the studies included comprised predominantly white women. Only two studies reported a more diverse sample [12, 29], which may be reflective of the wider population in the study area, but this information is not made explicit.

#### Proportion of eligible and willing participants recruited by method of referral

A further analysis of the proportion of eligible and willing participants by mode of referral showed that agency referrals resulted in a higher proportion of eligible and willing participants compared to self-referral. Table 4 shows the proportion of participants recruited vs participants who indicated interest via agency and self-referral. The average proportion of participants recruited into studies where agency referral was used was 74% while the average proportion of participants recruited into studies where self-referral was used was 40%.

**Table 4:** Proportion of agency referred and self-referred participants recruited

Studies	Proportion of agency referred participants recruited	Proportion of self-referred participants recruited
Bouwman et al., 2017	N/A	71%
Dickens et al. 2011	61%	N/A
Fokkema and Knipscheer 2007	68%	
Greenwald and Beery 2001	68%	N/A
Honigh-De Vlaming et al. 2013		48%
Ollonqvist et al. 2008	100%	N/A
Pitkala et al. 2009	N/A	15%
Slegers et al. 2008	N/A	24%
Average	74%	40%

## Discussion

Recruitment of older adults at risk of loneliness is an important, yet often overlooked topic. This review provides a comprehensive analysis of the recruitment techniques employed across studies aimed at preventing loneliness and social isolation. One of the key strengths of this review is the rigour and robustness of the research approach used to identify eligible studies.

There was a high level of heterogeneity between the studies included in terms of the design of the intervention, country and setting, and participants’ characteristics. The definition of ‘older’ adults varied considerably across most of the studies, with only an imprecise definition used in some cases. The age restrictions could have had an impact on the size of the target population.

The most common strategy for inviting people to participate in intervention studies were public-facing methods including mass media, advertisement in flyers, posters in community settings and local newspapers. The majority of participants identified this way were self-referred, and in many cases self-identified as lonely. This may be a potentially self-defeating strategy, as it inherently targets a population who may be more socially connected. In most cases where participants were invited via public facing methods, there was no data on the total number of people invited, so a response rate could not be calculated. Available data from two studies that relied on print media advertisement alone showed that 93 and 83% of the respondents who initially indicated an interest in the study were not enrolled to the intervention [11, 17]. It is not clear whether this difference is primarily based on failing to meet eligibility criteria or willingness to participate on the part of the respondents, or a combination of both factors.

Available data from studies that used referrals from agencies and organisations shows that about 60 to 100% of participants who were referred by agencies were eligible and willing to be enrolled into the study [20, 24,]. The findings from these studies demonstrates that referrals from key agencies could, therefore, be a more promising way of identifying eligible and willing older adults at risk of loneliness and social isolation. Given that older adults at risk of becoming socially isolated are more difficult to reach, researchers wishing to work with this group need to partner closely with organisations and agencies who are in contact with them [32]. Referrals from primary care were only used in a few studies. This may be an area for further development, as older adults tend to have relatively frequent consultations with General Practitioners. This is substantiated by findings from Hobbs et al. which showed a significant increase in GP consultation rates among older women aged 85 and above [33]. Referrals from other organisations who may have regular contact with older people such as pharmacists, social care or chiropodists were not used in this study and very few studies reported using referrals from public services. None of the studies conducted in the UK reported working with the Fire and Rescue Service to identify vulnerable older adults at risk of becoming socially isolated. This appears to be a missed opportunity given the increased collaboration between The Fire and Rescue Service and Health and Social Care in the UK to enable identification and referral of vulnerable groups. This has led to an expansion of the role of fire services to focus on wider health and wellbeing of individuals identified through the new Safe and Well visits [34]. Also, the wider social care workforce including care workers and others such as hairdressers, who have regular contact with older people in the community, may have a role to play in signposting and identifying older people a risk [32, 35].

Personalised travel planning interventions aimed at enabling people to choose more active means of travel, may as a by-product have the potential to identify those at risk. The implementation of this program involves approaching households in an area to provide information about opportunities for active transport. Evidence from the UK showed that staff members involved in the implementation were able to interact with older people with limited access to social opportunities [36].

Studies that combined multiple forms of recruitment that includes agency referral in combination with either print media, or mass-media advertisement showed more promising recruitment rates than those that relied on only one method of recruitment. For instance, a combination of multiple forms of recruitment including mass-media campaign, print media advert, and talks at municipal fairs could partially explain the high recruitment rates reported in the study by Honigh-De Vlaming and colleagues [30]. It is, however, important to recognise that using multiple forms of recruitment may be more time and resource consuming. Other methods of identification such as the use of questionnaires to determine people at risk of loneliness or social isolation are also quite resource intensive.].

The findings from this review are consistent with existing literature. A review of interventions to reduce loneliness and social isolation reported that ‘participants were identified from agency lists (GPs, social services, service waiting lists), or through mass media solicitation [6]. Three studies included in the review acknowledged a problem of self-selection [6]. The systematic review by Dickens at al (2011) compared how older adults at risk of isolation were targeted across intervention studies. The authors reported that, in most cases, assumptions about loneliness and social isolation were implied based on judgements of personal circumstances (e.g., widow) (*n* = 20) and fewer interventions relied on self/professional assessment (*n* = 12). The findings of our systematic review highlight the sparsity of information provided about the methods of identifying and recruiting eligible and willing participants. These factors limit the conclusions that can be drawn about the most effective methods.

### Limitations of the review

This review also has some limitations. One of the limitations is the lack of data needed to evaluate the cost-effectiveness of the various recruitment techniques. A cost-effectiveness analysis was outside the scope of this review and the majority of the studies included in the review did not provide the necessary data to evaluate and compare the cost-effectiveness of their recruitment strategies. Further research is needed to investigate and compare the cost-effectiveness of the recruitment strategies identified in this review.

Another limitation is the decision to include only studies from high-income countries. This decision was reached in order to reflect the particular challenges faced by researchers and practitioners in developed countries.

### Implications for research and practice

This study examined the various methods used to identify and recruit older people at risk of social isolation and loneliness into intervention studies. The findings from this study show that researchers and authors need to be more transparent in writing up the methods used to approach, assess and enrol older adults at risk of becoming socially isolated. None of the intervention studies included in this review justified their recruitment strategies. There was insufficient information on effective gender-based approaches, and strategies used to approach those from minority ethnic backgrounds. The heterogeneity of socially isolated older adults has been highlighted [37]. People from minority ethnic groups and older men are less likely to self-identify as being lonely or socially isolated. Given the challenges in this area, it would be helpful for researchers to provide more details of recruitment and retention procedures, including costs. This will enable best practice to be developed in this area. There is no ‘gold standard’ (such as a commonly used and validated framework or protocol) for the identification and recruitment of lonely or socially isolated older people. However, there are tools to assess various aspects of social relationships [38]. Some of these include the UCLA Loneliness Scale [39] and the de Jong Gierveld Loneliness Scale [40]. There are also guidelines and checklists to ensure transparency in conducting and reporting research studies. Examples of these include CONSORT guidelines for randomised trials [41] and the STROBE statement for observational studies [42]. The gap in reporting of the methods and processes identified in studies included this review may have an impact on the validity of recruitment strategies and therefore the outcomes of intervention studies, as those most in need of the intervention may not be included.

Findings from this study also have implications for intervention developers. Referrals by agencies and organisations such as GPs can potentially be a more effective means of identifying target participants. A report from the Campaign to End Loneliness called for a partnership between commissioners or service providers and staff at organisations and agencies experienced at working with people at risk of social isolation to target preventative effort more effectively [2]. There may be scope to broaden the range of agencies outside those in health and social care to include others who visit older people in their homes for other reasons such as the fire brigade.

## Conclusion

This review highlights a lack of evidence on the most effective methods of recruiting older people into research to tackle social isolation and loneliness. However, the findings suggest that a combination of two or more recruitment strategies that includes referrals from relevant agencies might be more effective than relying solely on public facing methods. This would, however, need to be considered in the light of available resources and time.

There is also a lack of information on the most cost-effective approaches. Future intervention studies should include more detail on the methods of recruitment used, and publish data on the effectiveness and cost-effectiveness of their recruitment strategies. The development of any recruitment framework should include primary care, other public services and community groups working with older people.

As well as informing the development of a robust system for recruitment of older people into research, the outcomes from this review can contribute to the development of a process for wider health and social care services to work together to identify those who may need support.

## Data Availability

The datasets used and/or analysed during the current study available from the corresponding author on reasonable request.

## Abbreviations

CONSORT: Consolidated Standards of Reporting Trials
GP: General Practice
RCT: Randomised Controlled Trial
STROBE: STrengthening the Reporting of OBservational studies in Epidemiology
